# Emerging insights into keratin 7 roles in tumor progression and metastasis of cancers

**DOI:** 10.3389/fonc.2023.1243871

**Published:** 2024-01-08

**Authors:** Hamed Hosseinalizadeh, Qusay Mohammed Hussain, Zahra Poshtchaman, Muhammad Ahsan, Ali H. Amin, Soroush Naghavi, Mahmood Khaksary Mahabady

**Affiliations:** ^1^ Department of Medical Biotechnology, Faculty of Paramedicine, Guilan University of Medical Sciences, Rasht, Iran; ^2^ Department of Pharmacy, Al-Noor University College, Nineveh, Iraq; ^3^ Department of Nursing, Esfarayen Faculty of Medical Sciences, Esfarayen, Iran; ^4^ Silesian University of Technology, Gliwice, Poland; ^5^ Zoology Department, Faculty of Science, Mansoura University, Mansoura, Egypt; ^6^ Student Research Committee, Iran University of Medical Sciences, Tehran, Iran; ^7^ Anatomical Sciences Research Center, Institute for Basic Sciences, Kashan University of Medical Sciences, Kashan, Iran

**Keywords:** Keratin 8 (KRT8), cytokeratin-7 (CK -7), keratin-7 (K7), epithelialmesenchymal transition (EMT), progression-free survival (PFS), microsatellite stability (MSS), microsatellite instability (MSI), Acetyl-CoA synthetase-2 (ACSS2)

## Abstract

Keratin 7 (KRT7), also known as cytokeratin-7 (CK-7) or K7, constitutes the principal constituent of the intermediate filament cytoskeleton and is primarily expressed in the simple epithelia lining the cavities of the internal organs, glandular ducts, and blood vessels. Various pathological conditions, including cancer, have been linked to the abnormal expression of KRT7. KRT7 overexpression promotes tumor progression and metastasis in different human cancers, although the mechanisms of these processes caused by KRT7 have yet to be established. Studies have indicated that the suppression of KRT7 leads to rapid regression of tumors, highlighting the potential of KRT7 as a novel candidate for therapeutic interventions. This review aims to delineate the various roles played by KRT7 in the progression and metastasis of different human malignancies and to investigate its prognostic significance in cancer treatment. Finally, the differential diagnosis of cancers based on the KRT7 is emphasized.

## 1 Introduction

A scaffold of actin microfilaments, microtubules, and intermediate filaments supports the cytoplasm of eukaryotic cells. Intermediate filaments, 10 nanometers in diameter, lie between the microfilaments (6 nanometers) and microtubules (23 nanometers) (1). Intermediate filaments (IFs) play a significant role in a multitude of biological processes, such as the positioning of organelles and protein targeting, the polarization of cells along an apical–basal axis, cellular motion, the determination of cell size, the synthesis of proteins, transmembrane transport, the progression of the cell cycle, cell death, and signal transduction. Keratins are the most diverse among IFs and belong to the type I (acidic) and type II (basic) IF proteins (1, 2).

KRT7 (Cytokeratin 7), belonging to the cytokeratin type II family, is predominantly expressed during the differentiation of simple and stratified epithelial tissues. The gene responsible for encoding KRT7 can be found in a specific region of chromosome 12q12-q13 (3). They not only contribute to the structural integrity of the cytoplasm but are also involved in various other biological processes. These include inhibition of interferon-dependent interphase, promotion of DNA synthesis in a possible cooperation with specific growth factors or hormones, initiation of translation, possibly through interaction with p150, the largest subunit of eukaryotic translation initiation factor 3 (eIF3), and interaction with G protein-coupled estrogen receptor 1 (GPER1), which activates several signaling pathways (4, 5).

In a physiological context, KRT7 expression is associated with normal proliferation, migration, and epithelial-mesenchymal transition (EMT), all of which play important roles in embryo development, immune system response, and wound healing. However, their higher expression in cancer is associated with increased proliferation, migration, and EMT. The anomalous expression of KRT7 is linked to tumor progression and metastasis, resulting from oncogenic and epigenetic events. The exact function of KRT7 in the progression and metastasis of tumors is not yet clearly understood. KRT7 is expressed in most cancers, except colorectal carcinoma, prostate cancer, renal cancer, thymic carcinoma, carcinoid, and Merkel cell carcinoma. Thus, it is utilized as a diagnostic marker in tumor pathology. Recent studies have provided evidence that KRT7 should be regarded as a marker and regulator of signal transduction in cancer cells (6, 7). The increase in expression of KRT7 during the process of EMT holds significant predictive value for various types of tumors, serves as a mere indication of metastasis and chemoresistance (6, 8).

​Monoclonal antibodies (MAbs) targeting keratin 7 have demonstrated efficacy as markers for distinguishing between various types of cancer. For instance, they have been utilized in the differentiation of mCRC (metastatic colorectal carcinoma), which is KRT20-positive and KRT7-negative, from primary ovarian carcinoma, which is KRT20-negative and KRT7-positive. Additionally, they have been applied to differentiate between primary liver adenocarcinomas, which are KRT7-positive, and metastatic liver adenocarcinomas, which are KRT7-negative (9, 10). Recent research has indicated that KRT7 plays a significant role not only in cellular proliferation, programmed cell death, and oncogenic metabolism but also functions as a critical modulator of the tumor microenvironment and immunological response (11). Elevation in KRT7 expression has been linked to a repressive immune microenvironment (11, 12).

Given the pivotal role played by KRT7 in the progression and metastasis of cancer, it is imperative to provide a comprehensive overview of the latest research findings on this protein. This comprehensive review delineates recent discoveries regarding the function of KRT7 in cancer progression and metastasis. Furthermore, this review accentuates the significance of KRT7 as a crucial modulator of the tumor microenvironment and immune response, and its involvement in cancer differentiation.

## 2 The prognostic significance of KRT7 and its role in EMT

Prognostic factors, which are measurable variables obtainable during diagnosis that are linked with disease-free or overall survival (OS), can be utilized to anticipate pre-treatment outcomes or the probability of disease recurrence. In cancer management, it is crucial to optimize treatment by employing prognostic factors. These factors are categorized into three groups: tumor-related, host-related, or environmental-related. Common prognostic factors for cancer comprise tumor histology, stage at diagnosis, tumor size, location, and patient age (13). KRT7 represents one of the most frequently activated oncogenes in various types of cancers. Its overexpression has been found to be a reliable indicator of unfavorable outcomes. Its involvement in cancer is widespread, as it promotes growth, cell cycle progression, metabolism, and survival. Consequently, several studies have focused on targeting KRT7 expression, either alone or in combination with other oncogenes, with promising results. This has rendered KRT7 a highly desirable therapeutic target for treating cancer. Recent research has highlighted the prognostic significance of KRT7 expression, demonstrating its crucial role as a prognostic marker for poor outcomes in cancer patients, regardless of age or other factors.

The BRAF V600E mutation is responsible for 8-10% of metastatic colorectal cancer (CRC) cases and is regarded as a poor prognostic factor. Median OS is less than 20 months for patients with the BRAF V600E mutation. The mutated BRAF gene translates to a protein that signals cells to divide uncontrollably, leading to tumors (14). There is emerging clinical evidence of significant heterogeneity in response to therapy in this patient group, despite the evidence of the prognostic significance of the BRAF V600E mutation. Only 5% of patients with BRAF V600E mutation CRC respond to a BRAF inhibitor (15). The findings of these studies imply that a more thorough stratification based on genomic factors must be explored when considering V600E BRAF mCRC as a distinct disease. Loupakis and colleagues employed a modern multivariate model, along with a clinically validated prognostic score, to provide insights into the prognostic significance of KRT7 in patients with BRAF (V600E)-mutated mCRC (16). The authors observed a tendency toward reduced OS in individuals with elevated CK7 expression, in comparison to those with low CK7 expression. Nonetheless, no significant disparities in progression-free survival (PFS) were detected. The current investigation has contributed novel insights into the impact of altered KRT7 expression on the survival of patients diagnosed with BRAF (V600E)-mutated mCRC and provided important and independent data that supplements the conventional clinical prognosticators, thereby enhancing the accuracy of patient prognosis assessment (16). Similarly, another study compared CK7 expression in BRAF-mutated CRC with MSS (microsatellite stability) and MSI (microsatellite instability). The results demonstrated a greater frequency of KRT7 expression in BRAF-mutated MSS CRC (39%) when compared with BRAF-mutated MSI CRC (6%) or BRAF wild-type MSS CRC (6%) (17). This study identified a correlation between altered CK7 expression, BRAF mutations, and microsatellite status in the context of CRC, which may present complications in the diagnosis of metastatic tumors (17). Acyl-CoA synthetase short-chain family member 2(ACSS2) is a vital enzyme in cancer metabolism, which during stress conditions, supplies tumor cells with Acetyl-CoA by taking up acetate as a carbon source. ACSS2 expression has been linked with poorer prognosis in certain malignancies (18). In relation to CRC, research has demonstrated the downregulation of ACSS2 expression. Kang et al. have shown that such downregulation is associated with a tendency to overexpress KRT7, and a decrease in expression of KRT20 (keratin 20) and CDX2 (caudal type homeobox 2)/CDX-3 (18). Thus, downregulation of ACSS2 expression could suggest a more hostile phenotype and unfavorable prognosis in CRC.

Pagliarulo et al. have suggested that the evaluation of KRT7 mRNA expression in circulating tumor cells (CTCs) among patients who have been diagnosed with urothelial bladder cancer prior to undergoing radical cystectomy (RC) or bladder removal surgery is a crucial tool for predicting patient prognosis and identifying potential candidates for systemic treatment (19, 20). The study revealed that the presence of KRT7 mRNA could serve as a prognostic indicator for unfavorable clinical outcomes. The detection rate of KRT7 mRNA-positive cells exhibited an upward trend with increased stages and lymph node status. In a cohort of 59 individuals, it was observed that a notable proportion of patients with non-muscle-invasive tumors, ranging from Ta, the lowest stage, to T1, exhibited a positivity rate of 26.3% for CK7. Furthermore, patients with pT2 tumors exhibited a positivity rate of 30.7%, while those with pT3 tumors exhibited a positivity rate of 38%. All of the patients with pT4 tumors were found to be CK7-positive. The evidence suggested that patients with a positive KRT7 status before RC were at a significantly higher risk of metastasis (7.8 times) and early death (5 times) compared to their CK7-negative counterparts. The hazard ratios were 8.77 and 5.2, respectively (19). Thus, the authors have furnished substantial evidence regarding the prognostic significance of detecting KRT7-positive cells prior to cystectomy and for the identification of covert cancer cases that would have otherwise remained undiagnosed. Nevertheless, it must be emphasized that the absence of sample size calculation renders it precarious to extrapolate their findings to a broader population with a greater number of variables (19). Koren et al. have demonstrated that the quantitative determination of mRNA expression of KRT7 in the peripheral blood of patients suffering from lung cancer is a highly sensitive technique for the molecular detection of circulating tumor cells (CTCs) that closely resemble A549 cells originating from lung adenocarcinoma (AC) (21). The study’s findings indicated that detecting a solitary tumor cell is possible in 2.5 milliliters of whole blood. Furthermore, it was observed that a definite association exists between the mRNA expression levels of KRT7 and the number of spiked tumor cells, and this relationship is consistently reproducible. These findings indicate that it is possible to detect KRT7-positive CTCs molecularly in whole blood, and this detection is dependent on the KRT7 mRNA expression level. The present study aimed to investigate the potential impact of KRT7 mRNA expression on the treatment response and prognosis of patients with advanced lung adenocarcinoma who underwent first-line platinum-based chemotherapy. However, the measured KRT7 mRNA levels did not show a significant relation to response to first-line platinum-based chemotherapy or survival in advanced lung cancer AC patients. Thus, further studies are necessary to ascertain the potential clinical significance of KRT7 mRNA expression in whole blood following chemotherapy (21).

**Figure 1:**
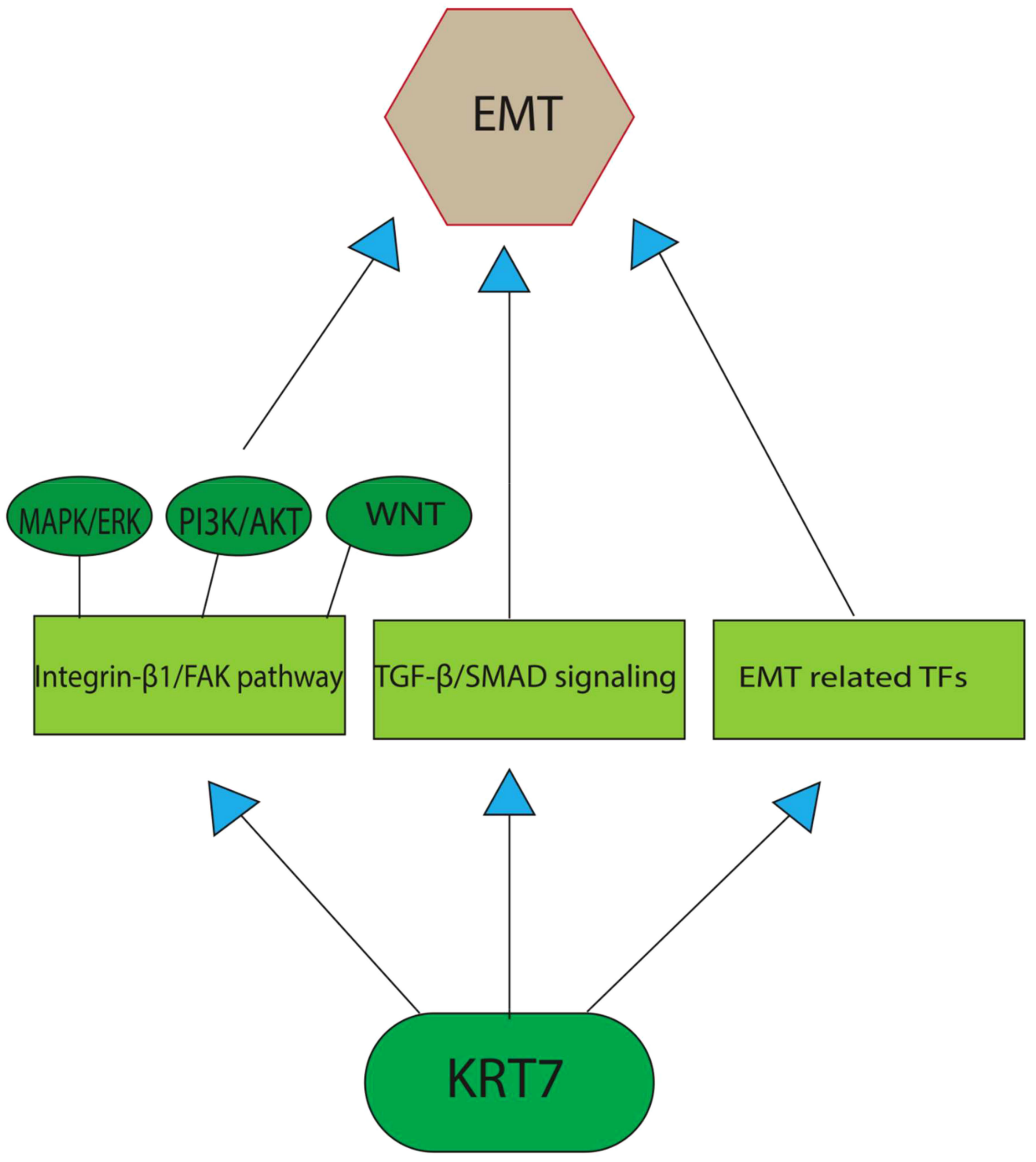
A graphical depiction of the primary pathways through which KRT7 enhances the process of EMT. TGF-β/Smad2/3 pathway, which is activated by KRT7, represents the primary pathway responsible for inducing EMT in cancer cells. The other potent pathway includes the activation of the integrin-β1/FAK pathway, the fundamental prerequisite for the activation of various EMT-promoting signaling pathways, such as MAPK/ERK, PI3K/AKT, and WNT. Recent studies also found a positive correlation between KRT7 expression and the transcription factors associated with EMT, such as Snail1, Snail2, Twist1, and Twist2, as well as mesenchymal markers MMP2, MMP9, Fibronectin, and Vimentin/CTRCT30.

The role of EMT in the tumorigenic process has been demonstrated in recent studies. EMT involves the acquisition of the mesenchymal phenotype by epithelial cells, resulting in significant remodeling of cell-cell and cell-extracellular matrix interactions (22). This process is characterized by cytoskeletal reorganization and the downregulation of certain epithelial markers, such as E-cadherin, claudins, and keratins, which consequently promote cell motility. However, it is important to note that only a subset of keratins is downregulated during EMT, as others, including KRT7 and KRT16, remain stable or even increase in expression (8, 23). To explore the involvement of cytokeratin in EMT, the researchers subjected epithelial cells to TGF-β1 (transforming growth factor beta 1)/DPD1 treatment *in vitro*. This resulted in a rise in the expression of various EMT factors, such as Snail1 (Snail Family Transcriptional Repressor 1) and ZEB1(zinc finger E-box binding homeobox 1)/TCF8, in a Smad2/3-dependent manner (24). Despite the expectation that cytokeratin would hinder EMT development, certain constituents of this group promote it during the early stages of TGF-1-induced EMT. An et al. have shown that the TGF-β/Smad2/3 pathway, which is activated by KRT7, represents the primary pathway responsible for inducing EMT in ovarian cancer. The positive correlation between the expression of KRT7 and TGF-1, ITG-1 (inhibition of thrombin generation-1), MMP2 (Matrix Metallopeptidase 2), and SMAD2 (SMAD Family Member 2), confirms its participation in the EMT and cellular migration through the TGF-β signaling pathway. The translocation of Smad2/3 to the membrane is facilitated by KRT7 activity, thereby enabling the phosphorylation of Smad2/3 through TGF-1 receptor kinase. However, the primary mechanism through which KRT7 promotes EMT instead of inhibiting it remains unclear. In contrast to the control group, it was observed that the knockdown of KRT7 resulted in a notable reduction in the levels of p-Smad2/3/Smad2/3 and vimentin, along with an elevation in the expression of E-cadherin. Furthermore, there was a positive correlation between the expression of KRT7 mRNA and other transcription factors associated with EMT, such as Snail1, Snail2, Twist1, Twist2, as well as mesenchymal markers MMP2, MMP9, Fibronectin, and Vimentin/CTRCT30 (25). Vimentin, classified as a type III intermediate filament, has been recognized as a potent promotor for EMT-mediated metastasis. While mesenchymal cells usually manifest minimal levels of vimentin expression, cancerous cells have a propensity to excessively express this protein, a phenomenon that has been correlated with increased tumor progression, invasion, and unfavorable prognosis. Nevertheless, the precise role of vimentin in cancer development remains unknown (26). Based on research findings, it has been observed that overexpression of KRT7 is linked to elevated expression levels of FAK (Focal Adhesion Kinase)/protein tyrosine kinase 2 (PTK2) and integrin-β1. This indicates that KRT7 might have a role in ECM (extracellular matrix) degradation in cancer cells, which in turn enhances their invasiveness. Integrins are a class of transmembrane receptors that are known to play a significant role in signal transduction, cell growth, differentiation, viability, death, invasion, and metastasis of cancer cells. Integrins can engage with ligands present in the extracellular matrix, ligands present on the outer surface of the cells, as well as soluble ligands. This process promotes the adhesion of cells to neighboring cells and the extracellular matrix (ECM). Subsequent to binding to the ECM, activation of integrins leads to their accumulation at the cell membrane level and subsequently activates their downstream targets, such as FAK and Src family protein tyrosine kinases (PTKs). The activation of the integrin-β1/FAK pathway is a fundamental prerequisite for the activation of various signaling pathways, such as MAPK/ERK, PI3K/AKT, and WNT. These pathways have been known to regulate EMT in different ways and have a significant impact on the aggressiveness of tumors (**Figure 1**) (27, 28). Additionally, the expression of MMP-9 and the activation of the integrin-β1/FAK pathway have been associated with cancer cell invasion, metastasis, and cell motility (29). MMP-9 is an endopeptidase dependent on zinc and has been implicated in proteolysis by cleaving various ECM components and non-ECM molecules. MMP-9 overexpression is a common occurrence in the majority of cancer types. Its involvement in the processes of invasion, angiogenesis, and metastasis has been well documented (30). These findings strongly suggest that KRT7 serves a crucial function in the initial and possibly other phases of EMT. Further investigations are required to determine whether this is a KRT7-specific effect or a property that is common to cytokeratins as a whole.

## 3 The role of KRT7 in metastasis

The expression of long non-coding RNA (lncRNA) keratin-7 antisense (KRT7-AS) depicts a downregulation in various cancer types, thereby emphasizing the tumor-suppressive role of endogenous KRT7-AS in multiple cancers (31). Breast and lung cancers have demonstrated a prevalent KRT7-AS deficiency, and low levels of KRT7-AS indicate a poor prognostic factor in breast cancer (BC). Cellular investigations reveal that the silencing of KRT7-AS in lung cancer cells upregulates oncogenic Keratin-7 levels, which augment tumorigenesis while reducing cancer apoptosis (31). On the other hand, overexpression of KRT7-AS (by small molecule berberine) inhibits tumorigenesis and sensitizes cancer cells to platamin, an anti-cancer drug. Retinoid X Receptor Alpha (RXRα), a transcription factor, is crucial for KRT7-AS expression. Mechanistically, KRT7-AS lowers the levels of oncogenic Keratin-7 and significantly elevates the amounts of PTEN, a key tumor suppressor, in cancer cells by inhibiting the degradation of PTEN via the ubiquitin pathway (by binding directly to PTEN via its GGCAAUGGCGG motif) (31).

**Figure 2:**
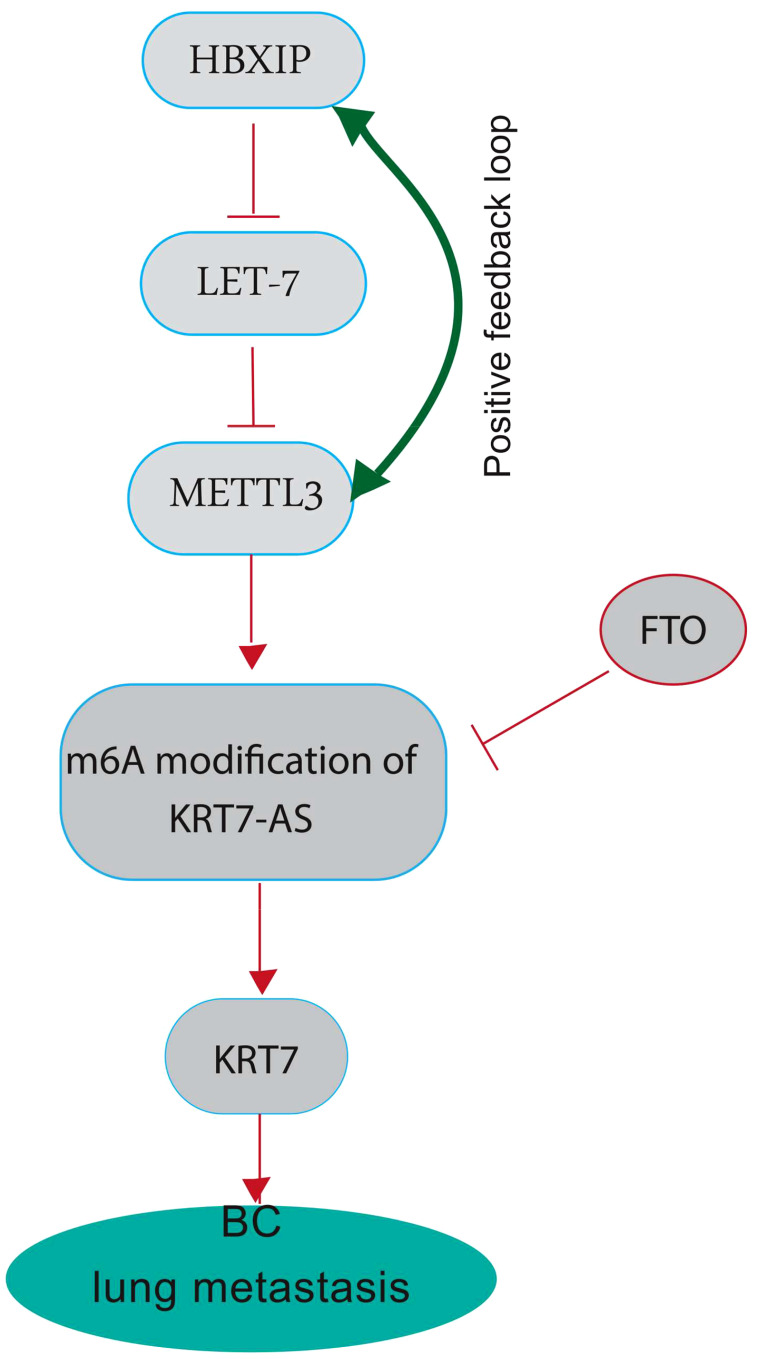
A graphical highlighting the impact of KRT7 on breast cancer lung metastasis. METTL3 plays a crucial role in several human cancers, mainly through its m6A RNA methyltransferase activity. METTL3 can methylate long non-coding RNA Keratin7-antisense (KRT7-AS) in its CDS regions (the coding sequence) to increase its expression, forming a duplex with KRT7 mRNA and leading to increased stability and translation. High KRT7 expression is associated with breast cancer lung metastasis. HBXIP inhibits miRNA let-7, a negative regulator of METTL3, thereby maintaining METTL3 expression. METTL3 methyltransferase activity induces HBXIP expression, indicating a positive feedback loop. FTO, also known as the “eraser of m6A,” catalyzes m6A demethylation and appears to have a tumor-suppressive effect by demethylating KRT7-AS and decreasing its expression. FTO shows decreased expression in BC cells during LM.

The modification of N6-methyladenosine (m6A) is a prevalent epigenetic regulation in mammals and has been shown to have a substantial impact on the metastasis of various cancers, specifically BC (32). The effects of m6A are extensive, influencing numerous RNA metabolic processes, such as mRNA degradation, stability, and translation. There is mounting evidence indicating that methyltransferase-like 3 (METTL3) is a critical factor in multiple human cancers, primarily due to its activity as an m6A RNA methyltransferase (33). The upregulation of METTL3 expression is dependent on the expression of the hepatitis B X-interacting protein (HBXIP). HBXIP prevents miRNA let-7 g, which acts as a negative regulator of METTL3, from inhibiting METTL3, thus maintaining its expression and accelerating cell proliferation and metastasis in breast cancer. The induction of HBXIP expression is caused by the methyltransferase activity of METTL3, thereby suggesting the existence of a positive feedback loop (34). Recent investigations have revealed that METTL3-mediated KRT7 m6A methylation is essential for BC lung metastasis (LM). Chen and colleagues aimed to illustrate the epigenetic reprogramming related to m6A in BC cells during LM (25). Their study revealed that the augmented expression of METTL3 can methylate KRT7-AS in its CDS regions (coding sequence), thus elevating its expression. The resultant duplex formation of KRT7-AS with KRT7 mRNA leads to increased KRT7 mRNA stability and translation. Conversely, they noted a decline in the expression of FTO (Fat Mass and Obesity-Associated Gene) in BC cells during LM (25). FTO also referred to as the “eraser of m6A,” functions in a Fe (II) - and -ketoglutarate-dependent manner to catalyze m6A demethylation. FTO appears to have a tumor-suppressive effect by demethylating KRT7 and reducing its expression (**Figure 2**) (35). The increased expression of KRT7 mRNA could be attributed to the protection of the 3’ end of KRT7 mRNA from microRNAs (miRNAs) and RNA exosome (the main 3’-5’ eukaryotic RNA-degradation complex) targeting. Studies on gastric cancer (GC) have revealed that the OL region of KRT7-AS, which corresponds with KRT7, exerts a significant protective effect. It is important to note that solely the OL region or the complete length of KRT7-AS exhibited an augmentation in KRT7 protein levels under conditions of enforced overexpression, whereas the non-OL region failed to show such outcomes (36). Furthermore, the data sets showed that m6A residues in CDS have a positive effect on the translation by recruiting multiple reader proteins such as IGF2BP1/HuR and resolving mRNA secondary structures (25, 37). IGF2BP1 (insulin-like growth factor 2 mRNA-binding protein 1)/ZBP1 is considered a promising m6A reader that facilitates the expression of methylated KRT7 mRNA. LINC00266-1 encodes a 71-aa peptide known as RNA-binding regulatory peptide (RBRP), which mainly interacts with IGF2BP1 and amplifies its recognition of m6A on RNAs, such as KRT7 mRNA. This interaction enhances the stability and level of KRT7 mRNA by increasing the recruitment of RNA-binding proteins, such as HuR (human antigen R; encoded by the Elavl1 gene) (38). Decreased FTO expression also induces methylation of KRT7 mRNA at A950 and may trigger the recruitment of a complex called YTHDF1/eEF-1 to facilitate translation elongation. YTHDC2 (YTH N6-Methyladenosine RNA Binding Protein C2), an m6A reader that contains an RNA helicase, binds to m6A residues near the proximal 3’UTR and facilitating the rate-limiting step of translation, which occurs during initiation, by enhancing their association with polysomes, likely through a mechanism involving mRNA looping (39).

Wang et al. found that the expression of KRT7 and KRT7-AS increases in CRC-LM (40). They also found that Fusobacterium nucleatum (Fn), a CRC-enriched gram-negative anaerobic bacterium associated with poor prognosis and chemoresistance in humans, greatly increases KRT7-AS expression in CRC cells. The KRT7-AS targets KRT7 and plays a crucial role in the augmented expression of KRT7 within CRC cells (41). Upon inhibition of KRT7-AS or KRT7, a significant decrease in cell migration was observed in HCT-116, a human colorectal cancer cell line, when subjected to Fn infection. On the contrary, overexpression of KRT7-AS or KRT7 in HCT-116 or LoVo (a colon adenocarcinoma cell line) demonstrated a significant augmentation in cell migration (40). The TLR4/MYD88/NF-κB signaling pathway is the mechanism by which Fn infection triggers upregulation of KRT7-AS. Toll-like receptor 4 (TLR4)/CD284 is the primary sensor that recognizes various microbial components and triggers innate immune responses (42). The pathway of NF-κB can be stimulated through TLR4 to regulate host cellular processes subsequent to exposure to diverse microbial pathogens, such as the case of Fn infection (40, 43). Phospho-p65 (also known as RelA and NFKB3) is upregulated and transported to the nucleus, where it binds to the promoter region of KRT7-AS and stimulates its expression (40). Similarly, Fei et al. have shown that CRC cells expressing KRT7 have a notable potential for invasiveness and metastasis to lymph nodes. They demonstrated a correlation between KRT7 expression and localization (with more cases of KRT7-positive cells in the ascending colon), differentiation (with poorly differentiated cells having more KRT7 expression than moderately or highly differentiated cells), and Duke’s stage of CRCs. Cells positive for KRT7 were predominantly situated at the periphery of cancer nests, at the invasion front, within tumoral buds, and within intravascular tumor emboli (44). Meanwhile, SANO et al. identified KRT7 as a marker gene for esophageal squamous cell carcinomas (ESCCs) with significant lymph node metastasis (45). They also discovered that Forkhead box protein A1 (FOXA1)/Transcription Factor 3A is the upstream transcriptional regulator of KRT7 and directly stimulates KRT7 transcription, which implies that FOXA1 is crucial for cancer cell metastasis (45, 46). The treatment of ESCCs with FOXA1 siRNA led to a decrease in mRNA levels of KRT7 and LOXL2, a protein from the lysyl oxidases-like 2 family. In ESCCs with metastatic lymph nodes, FOXA1 is known to stimulate the production of not only KRT7 but also LOXL2. LOXL2 has a substantial impact on diverse intracellular processes, including EMT, and is associated with an unfavorable prognosis and an increased risk of distant metastases (47). However, FOXA1 expression was only detected in 60% of ESCCs with metastatic lymph nodes, indicating that other transcription factors must activate KRT7 and genes associated with metastasis in the remaining 40% of cases. Le et al. conducted an assessment of the inhibitory impact of sennoside A (SA)/Senokot on the metastasis of hepatocellular carcinoma (HCC) cells both *in vitro* and *in vivo* and examined alterations in the transcriptome of HCC cells upon exposure to SA (48). SA is a primary compound of anthraquinone/anthracenedione extracted from Rheum officinale (Da Huang) and is utilized in several countries as a stimulant or irritant laxative, as a herbal medicine for weight loss, or as a dietary supplement. The findings indicated a noteworthy decrease in intrahepatic metastasis of HepG2 cells, which is a type of human liver cancer cell line, after the administration of SA. Transcriptome analysis led to the identification of 9 potentially HCC metastasis-related genes that may potentially be subject to regulation by SA in HCCs. Among these genes, KRT7 was downregulated in cell lines upon treatment with SA. Thus, SA has been identified as a natural non-specific inhibitor of KRT7 expression (48).

The transcription factor FoxM1 (Forkhead Box M1), which is also referred to as M-Phase Phosphoprotein 2, plays a crucial role in mitosis and chromosome segregation. It operates as a significant regulator of the cell cycle by controlling the advancement from G1 to the S phase (49). The overexpression of this gene has been associated with various types of tumors and is connected to metastasis, progression, and unfavorable prognosis in individuals who have been diagnosed with cancer (49–51). Therefore, there has been a significant focus on developing drugs that target FoxM1 for antitumor therapy, including purinol, RCM-1, FDI-6, and Siomycin/Sporangiomycin (52, 53). However, there is still a need to fully understand the molecular network downstream of FoxM1. In a recent study, Zhang et al. explored the downstream molecular network of FoxM1 in ovarian cancer and identified KRT5 and KRT7 as downstream target genes of FoxM1 (7). To verify this finding, FoxM1 expression was down-regulated and the impact on KRT5 and KRT7 expression levels was evaluated in SKOV3 cells, a type of human ovarian cancer cell line. It was observed that the suppression of FoxM1 expression dramatically disrupted the expression of KRT5 and KRT7 and inhibited SKOV3 cell migration (7). Their findings suggest that FoxM1 regulates KRT5 and KRT7 via AP2 (Adaptor Protein 2(,) a superfamily of transcription factors, and that the binding site of FoxM1 to KRT5 and KRT7 is situated in the exons rather than the usual promoter region. This suggests that FoxM1 regulates the expression of these two genes using an unknown mechanism (7). Thereby, FoxM1-KRT5/7 has the potential to serve as a new and innovative diagnostic marker as well as a therapeutic target for ovarian cancer.

The Wilms Tumor protein 1 (WT1) has demonstrated a notable impact on the expression of KRT7. Wang et al. carried out a comprehensive investigation on pancreatic cancer and found that WT1, a transcription factor, interacts with the KRT7 gene, leading to its expression by binding to a specific site in the 562-570 bp region upstream of the KRT7 promoter (54). The researchers also found that the levels of KRT7 expression were significantly elevated in tumor tissues compared to the surrounding tissues. Elevated KRT7 expression demonstrated a statistically significant association with augmented tumor size and advanced TNM stage. Furthermore, a negative association was detected between KRT7 expression and miR-216a, while a positive correlation was observed with WT1. The miRNA-mRNA regulatory network suggested miR-216a as a possible modulator of WT1 (54). By regulating the transcriptional expression of growth factor genes, the WT1 transcription factor could enhance cell proliferation, promote tumor cell infiltration, and facilitate metastasis (55). Jiang et al. compared whole genome expression of primary and metastatic sarcomas and subsequently identified potential prognostic biomarkers for tumor metastasis and therapeutic targets (56). Sarcomas, rare malignant tumors originating mainly in tissues like bone or muscle, are primarily treated with surgical resection and radiation therapy (57). However, sarcoma metastasis frequently occurs in the lungs, accounting for approximately 35-40% of sarcomas, and is often unresponsive to systemic therapy (57). The study identified KRT7 and MUC1 (Mucin-1)/CD227 as validated target genes through the list of genes upregulated in metastatic tissues. Patients with higher expression of KRT7 or MUC1 had a significantly poorer overall survival compared to those whose metastatic tumors exhibited lower expression levels of these genes. Interestingly, sarcoma cell migration was inhibited *in vitro* by siRNA knockdown targeting KRT7 and MUC1 (56). **Table 1** summarized sites of metastasis for primary tumors exhibiting KRT7 overexpression.

**Table 1:** Metastasis site for primary tumors exhibiting KRT7 overexpression.

Primary tumors	Metastasis sites	KRT7 positivity	Reference
pancreatic ductal adenocarcinoma	lymph	66.7% (18 out of 28 patients) of lymphatic metastasis	(11)
prostate cancer	bone metastasis	6.3% (18 out of 285 patients) of metastatic	(58)
cervical adenocarcinoma	lymph node	33 out of 126 of lymph node metastasis	(59)
Breast Cancer	Brain	95.7% (45/47) of breast cancer metastases to the brain	(60, 61)
colorectal cancer	lung	88% (21 out of 24) of metastatic colorectal cancers	
colorectal cancer	lymph node	4.1% of lymph node metastasis	(40, 62)
sarcoma	Lung	22 out of 30 (73%) of metastatic sarcomas	(56)
Esophageal squamous cell carcinomas	lymph node	15 out of 30 (50%) of metastatic ESCC	(45)

## 4 KRT7 overexpression is linked to immune suppression

Recent studies have demonstrated that KRT7 not only contributes significantly to cell proliferation and cancer metabolism, but also serves as a prominent regulator of the tumor microenvironment)TME)and immune response. According to Song et al., KRT7 expression is positively linked to the expression of S100A2 (S100 Calcium Binding Protein A2)/CAN19 (11). S100A2 is a member of the S100 protein family, a group of calcium-binding elongation factor (EF)-hand proteins that are highly conserved (12). The abnormal expression of S100A2 has an impact on various cellular physiological processes such as calcium homeostasis, enzyme activity, and protein phosphorylation. S100A2 is frequently overexpressed in different types of tumors and is often linked to tumor development (63). Furthermore, it has been discovered that S100A2 contributes to tumor growth by regulating the tumor microenvironment and evading the immune system. The heightened expression of S100A2 was linked to a decline in the percentages of CD8+ T cells, resting memory CD4+ T cells, and activated NK cells, while the proportions of M0 macrophages and activated dendritic cells increased (64). Among them, CD8^+^T cells, which have demonstrated the greatest efficacy in tumor eradication (65, 66), exhibited a marked reduction in the high S100A2 expression group, providing a partial explanation for the unfavorable prognosis observed in patients with high S100A2 expression (64). In addition, M0 macrophages were found to correlate with inferior prognosis across multiple cancer types (67).

**Figure 3:**
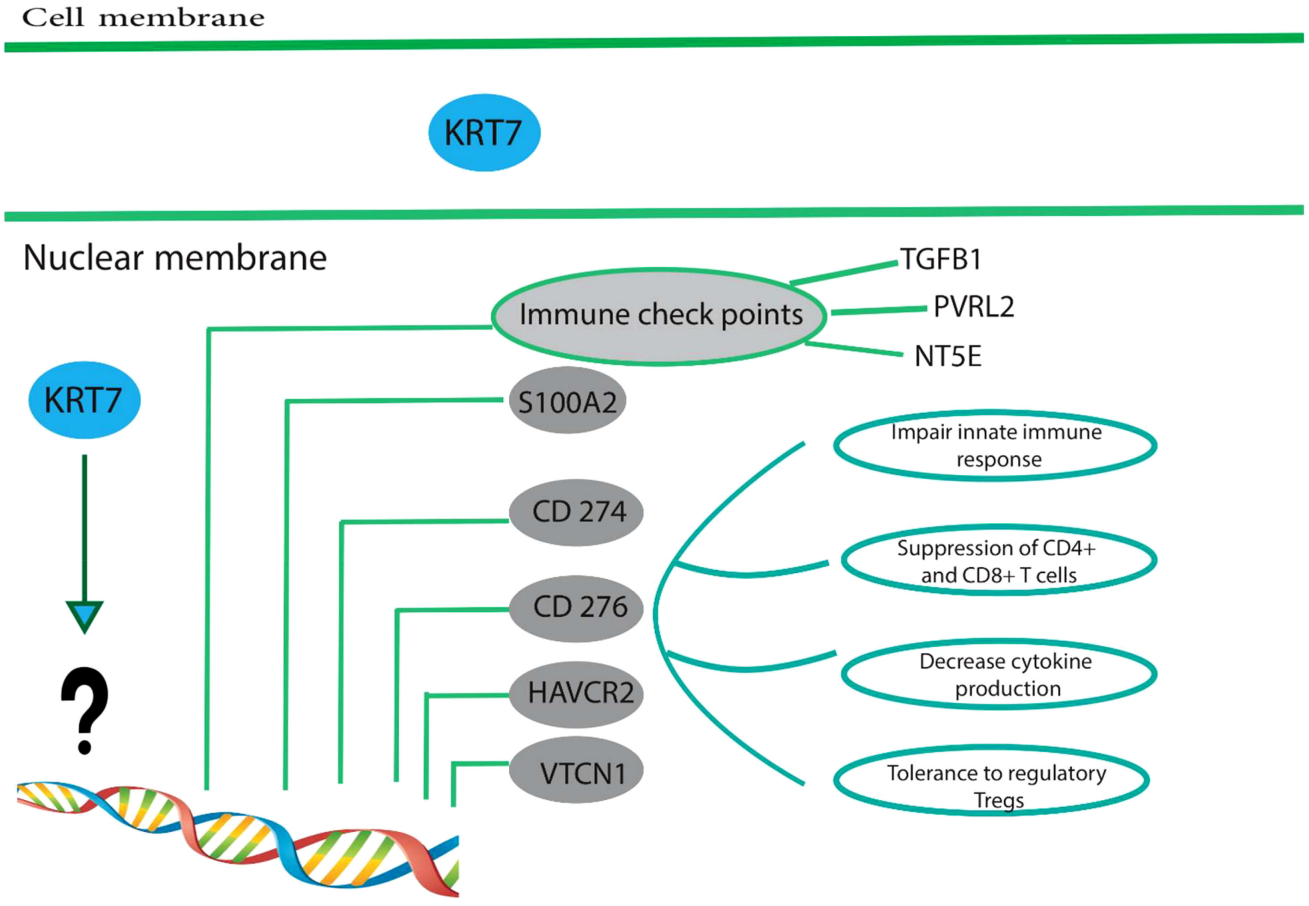
High expression of KRT7 is associated with a suppressive immune microenvironment. Overexpression of KRT7 in pancreatic cancer is related to the expression of S100A2, CD274, CD276, HAVCR2, and VTCN1.KRT7 expression also positively correlated with the expression of four common immune checkpoints in patients with adenocarcinoma of the pancreas, namely TGFB1 (CED), PVRL2 (Nectin-2), and NT5E (CD73). However, it is still unknown how KRT7 increases the expression of above-mentioned genes and needs further studies. These immune checkpoints are frequently upregulated in many different tumor types and are often associated with (1) impaired innate immune response, (2) suppressed CD4+ and CD8+, (3) decreased cytokine production and (4) tolerance to regulatory T cells. Therefore, tumor cells establish a particular condition called suppressive immune microenvironment suitable for tumor development and progression.

A study has demonstrated that the upregulation of KRT7 in pancreatic cancer is associated with the expression of immune checkpoints, including CD274, CD276, HAVCR2 (hepatitis A virus cellular receptor 2), and VTCN1 (**Figure 3**) (64). CD274, which is also referred to as B7-H1 or PD-L1, encodes a protein found on the cell membrane. This protein binds to the programmed cell death protein 1 (PD-1) on effector T cells and acts as an immunosuppressive agent by transmitting signals that suppress the effector T cells. It has been determined that for tumor cells to avoid immunosuppression, the PD-L1 protein must be expressed on their surface (68). These immunosuppressive signals impede T-cell activation and cytokine production (68). CD276/B7-H3 is a transmembrane glycoprotein of type I that is encoded by chromosome 9 in mice and chromosome 15 in humans. It is a member of the B7 and CD28 family, which are immunoregulatory molecules involved in T cell co-stimulation and co-inhibition (69). In the context of anti-tumor immunity, CD276 plays a dual role, acting as a T-cell stimulator to enhance T-cell activity, or as an inhibitor of T-cell function, thereby promoting tumor proliferation and invasion. It is notably overexpressed in various human solid cancers and is frequently associated with unfavorable clinical outcomes (69). Du and colleagues showed in their study that CD276-targeting chimeric antigen receptor (CAR) T cells effectively suppressed the development of PDAC (Pancreatic ductal adenocarcinoma), both *in vitro* and *in vivo* (70). HAVCR2, also identified as T cell immunoglobulin mucin 3 (TIM3) or CD366, encodes the protein TIM-3, a type I transmembrane protein with co-inhibitory properties (71). Tim-3 serves as a co-inhibitory receptor and is expressed on CD8+ T cells, Th1 CD4+ T cells, NK cells, Foxp3+ regulatory T (Treg) cells, and innate immune cells such as DCs and macrophages (72). Upon interaction with its ligand(s), Tim-3 has been observed to exert inhibitory function, leading to suppression of their responses and inducing the expression of cytokines like TNF-a and INF-γ. Because of its potential in cancer immunotherapy, Tim-3 has gained prominence as a potential candidate (71, 73). VTCN1 (V-Set Domain Containing T Cell Activation Inhibitor 1)/B7-H4 is a member of the CD28/B7 family of immune-inhibitory molecules. This molecule is present on the surface of APCs (Antigen-Presenting Cells) and interacts with ligands that attach to receptors on the surface of T cells, thus playing a crucial role in regulating T cell activation (74, 75). The higher expression of the VTCN1 protein is observed in various cancers. Studies have indicated that this increased expression is associated with the exhaustion and functional impairment of CD4+ and CD8+ T cells, and the formation of alloreactive cytotoxic T lymphocytes (CTLs). In preclinical models, myeloid dendritic cells, macrophages, and monocytes have been found to promote the expression of B7-H4 by IL-6 and IL-10 (74, 75).

Similarly, Li and colleagues have demonstrated that the expression of KRT7 is significantly and positively associated with four common immune checkpoints, namely TGFB1 (Transforming Growth Factor Beta 1)/CED, Nectin-2 (PVRL2), and NT5E (CD73) in patients diagnosed with adenocarcinoma of the pancreas (6). The regulation of endogenous antitumor immunity is controlled by immune checkpoints, and KRT7 can play a crucial role in influencing the antitumor immune response by upregulating the expression of immune checkpoints. Hence, the activation of the immune response and the consequent promotion of the survival of pancreatic cancer patients may be facilitated by the blocking of immune checkpoints. These findings suggest a correlation between KRT7 expression and immunosuppression within the microenvironment. Furthermore, cancer cells that exhibit heightened KRT7 expression may display heightened resistance to antitumor immunity. However, it is imperative to conduct further *in vitro* and *in vivo* investigations to determine the exact mechanism by which KRT7 regulates cancer immunity.

Studies demonstrated a significant association between KRT7 expression and immune infiltration of tumor immune cells into the TME (6). Tumor-infiltrating immune cells (TIICs) are essential constituents of the TME and have been utilized to prognosticate and treat cancer patients (76). Li et al. found that patients with high KRT7 expression in PAAD samples had lower proportions of cytotoxic T (Tc), mucosa-associated invariant T (MAIT), natural killer (NK), gamma delta T (Tgd), CD4+T, and CD8+T cells, compared to those with low KRT7 expression. On the contrary, it was found that the proportions of naive forms of cytotoxic T cells (CD8+), T helper 2 (TH2) cells, natural killer T (NKT) cells, dendritic cells (DCs), and monocytes within the KRT7 low expression cohort were relatively higher than those in the KRT7 high expression group (6, 66). CD8+ cytotoxic T cells play a crucial role in the immune response to cancer, as they are the main functional cells that can recognize and directly inhibit tumor cells, thus hindering their growth (77). Through the secretion of perforin, granzymes, and other cytotoxic factors, these cells can effectively kill tumor cells (78, 79). Studies have demonstrated that low tumor infiltration of CD8+ cytotoxic T cells in pancreatic cancer is indicative of poor overall survival and is a robust predictor of unfavorable clinical outcomes. Tgd cells, which are one of three immune cells expressing antigen receptors, promote effective cytotoxicity against numerous cancer cells and act as a bridge between innate and adaptive immunity (80, 81). Similarly, Song et al. found that increased KRT7 expression was significantly related to higher infiltration of memory B-cells, M0 macrophages, and activated mast cells, while the infiltration of naïve B-lymphocytes and monocytes was lower (11). Thus, alterations facilitated by KRT7 in the extent of immune cell infiltration, notably T cells, are believed to be intimately linked to the disruption of regular immune activity, consequently influencing the tumor microenvironment via the means of immune suppression.

## 5 Differentiate diagnosis of cancers via KRT7

The differentiation between primary cancer and metastatic carcinoma poses a typical clinical dilemma, and determining the site of origin can present a challenging task for the clinician. This distinction holds immense significance as the staging, treatment, and prognosis vary significantly between the two types of cancer (9). Despite the unfavorable prognosis for the majority of patients with metastatic carcinoma, a select group of patients may still be eligible for specific therapies. Therefore, it is crucial to employ all possible means to distinguish between primary cancer and metastatic carcinoma (9, 82). Recent studies have demonstrated that immunostaining for KRT7 and 20 can effectively differentiate primary ovarian carcinomas from metastatic carcinomas originating from the gastrointestinal tract and pancreas, which clinically resemble primary ovarian carcinoma (83). Adenocarcinomas of the colon and endometrial carcinomas are the most common cancers that metastasize to the ovaries and bear a resemblance to primary ovarian tumors (84). Wauters et al. have demonstrated that in 94% of cases, metastatic colorectal carcinomas are diffuse and exhibit strong KRT20 positivity while being KRT7 negative. However, certain studies have indicated KRT7 positivity in 20-30% of cases. In contrast, primary ovarian carcinomas exhibit an opposite immunophenotype, except for mucinous adenocarcinomas of the ovary, which frequently exhibit both markers. As a result, a CK7/CK20 immune profile may prove useful in differentiating primary ovarian carcinomas from carcinoma metastases to the ovary (83–85). It is important to note that adenocarcinomas of the small intestine, stomach, and appendix may exhibit CK7 positivity. Therefore, careful analysis of immunostaining for CK7 and CK20 is necessary, with consideration of all clinical information, while acknowledging that no tumor demonstrates absolute consistency in its staining with these markers. Hass and colleagues utilized oligonucleotide arrays to differentiate and identify potential biomarkers of intrahepatic cholangiocarcinoma (ICC) and colorectal metastases (86). Through microarray analysis, numerous dysregulated genes were identified in ICC, with KRT7 being overexpressed in over 80% of ICC cases. Interestingly, KRT7 was significantly downregulated in the analyzed tumor probes from metastatic adenocarcinomas, emphasizing the importance of KRT7 in distinguishing primary from metastatic adenocarcinomas of the liver in clinical practice (86). Roy et al. aimed to differentiate primary bladder adenocarcinoma (PBA) from metastatic colon adenocarcinoma (MCA), a challenging diagnostic and clinical issue (87). A group of antibodies comprising β-catenin, E-cadherin, CK7, and CDX-2(Caudal Type Homeobox 2) was utilized to differentiate PBA from MCA. The findings revealed that only 16% of PBA (less than 10% of stained cells) and 75% of MCA had strong nuclear with cytoplasmic membrane staining of β-catenin. Although both PBA and MCA showed abnormal nuclear E-cadherin staining, the former was more frequently affected. CDX-2 expression was documented in all cases of MCA and 83.3% of PBA. A significant number of PBA cases (one-third) and MCA cases (8.3%) exhibited strong CK7 expression, suggesting a limited ability to distinguish PBAs from secondary bladder adenocarcinomas based on CK7 and CDX-2. Despite the difficulty in clinicopathologic differentiation between PBAs and MCAs, as highlighted by this study, the small cohort size was a significant limitation. Therefore, more extensive studies are required to support the above conclusion (87).

Verdu et al. conducted a comparison between clinicopathologic features and molecular profiles of colorectal micropapillary carcinoma and conventional CRC (88). Colorectal micropapillary carcinoma, which is a rare and aggressive form of CRC, has a significantly lower survival rate and more lymph node metastases than conventional CRC. In terms of immunohistochemical profile, KRT7 expression was more prominent in micropapillary colorectal carcinomas (17%) than in conventional colorectal adenocarcinomas (3%). The expression of KRT7 was noted at the outermost regions of the tumor, which corresponds to the transition zones from glandular to micropapillary carcinoma patterns (88). This finding holds significant importance in the examination of CRC, as higher KRT7 expression is linked to micropapillary adenocarcinomas and not conventional colorectal adenocarcinomas. This distinction allows for more precise treatment options for CRC patients and aids in carefully assessing the extent of resection due to the heightened risk of lymph node metastasis (89). In a similar study, Szponar et al. demonstrated that expression of KRT7 and WT1 could distinguish precursor lesions of Wilms tumor from papillary renal tumor) pRCT (and mucinous tubular and spindle cell carcinoma) MTSCC (, a rare form of renal cell carcinoma. All precursor lesions associated with Wilms tumor were positive for WT1, while those associated with pRCT and MTSCC were negative. KRT7 positivity was found in 69-80% of lesions associated with pRCT and MTSCC, but not in WT-associated lesions (90).

Ichimi et al. have exhibited that there is a noticeable rise in the expression of KRT7 mRNA in bladder cancer (BC) in comparison to normal bladder epithelium (NBE). Furthermore, they have observed noteworthy inverse associations between the mRNA expression of KRT7 and the expression of miRNAs (91). Using BC miRNA expression signatures, they have identified a group of six downregulated miRNAs (miR-145, miR-30a-3p, miR-133a, miR-195, miR-125, and miR-199a) with tumor suppressive activity. According to their BC mRNA profile, they have predicted KRT7 as a common target of these downregulated miRNAs, indicating that multiple target genes are regulating KRT7 mRNA. To determine if these miRNAs reduce KRT7 expression, they have transfected the six miRNAs into KK47 cells (an established cell line of urinary bladder cancer) that overexpressed KRT7 mRNA. Following a transfection period of either 24 or 48 hours, there was a notable decrease in the expression of KRT7 mRNA. Specifically, the transfection of miR-199a was found to result in a significant reduction of nearly 50% in KRT7 mRNA expression when compared to the control. These results suggest that KRT7 expression may serve as a distinguishing factor in recognizing cancerous cells from healthy ones. Additionally, it implies that miR-145, miR-30a-3p, miR-133a, miR-195, miR-125, and miR-199a could potentially be employed as accurate diagnostic biomarkers and promising candidates for BC gene therapy (91). Mouteira et al. studied transitional cell carcinoma of the bladder by immunohistochemical analysis using monoclonal antibodies against KRT7 and KRT20 to identify metastatic carcinoma of bladder origin (92). Their findings revealed that all instances of transitional cell carcinoma of the bladder displayed KRT7 expression, while 75% tested positive for KRT20. Consequently, the Ck 7+/Ck 20+ immunophenotype holds great specificity for transitional cell carcinoma of the bladder, thus indicating its usefulness in identifying metastatic carcinoma of the bladder (92). The following **Table 2** summarizes the role of KRT7 expression in distinguishing between primary cancer and metastatic carcinoma.

**Table 2:** The potential of KRT7 expression in distinguishing between primary cancer and metastatic carcinoma.

Primary Cancer	KRT7	Metastatic carcinoma	KRT7	Application	Reference
Primary ovarian carcinomas	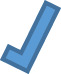	Metastatic carcinomas originating from the GI tract and pancreas	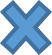	Useful in differentiating primary ovarian carcinomas from carcinomas metastases to the ovary	(83)
Intrahepatic cholangiocarcinoma	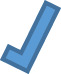	Colorectal metastases	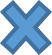	Distinguishing primary from metastatic adenocarcinomas of the liver in clinical practice	(86)
Primary bladder adenocarcinoma	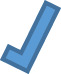	Metastatic colon adenocarcinoma	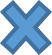	Differentiation between primary bladder adenocarcinoma and Metastatic colon adenocarcinoma	(87)
Colorectal micropapillary carcinoma	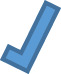	Conventional CRC	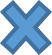	Distinguishing Colorectal micropapillary carcinoma from conventional CRC in clinical practice	(88)
Precursor lesions of Wilms tumor	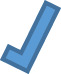	Papillary renal tumor and mucinous tubular and spindle cell carcinoma	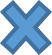	Distinguishing precursor lesions of Wilms tumor from Papillary renal tumor and mucinous tubular and spindle cell carcinoma	(90)
Bladder cancer	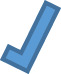	Normal bladder epithelium	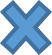	Useful in distinguishing bladder cancer from healthy ones	(91)
Transitional cell carcinoma of the bladder	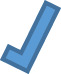	Metastatic carcinoma of bladder origin	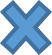	Useful in identifying metastatic carcinoma of the bladder	(92)

## 6 Conclusion

Although research has extensively examined KRT7 proteins in various malignancies, still, yet to design small molecule inhibitors that specifically target the KRT7 and interfere with its activity. Nevertheless, the use of noncoding RNA-based therapeutics has shown great promise for the development of targeted treatments for KRT7-over-expressing malignancies. In both *in vivo* and *in vitro* studies, miRNAs and small interfering RNAs (siRNAs) have been used to selectively suppress the expression of KRT7 mRNAs through the mechanism of RNA interference (RNAi), providing a remarkable therapeutic tool for targeted therapies and precision medicine. Through our review, we have established that the levels of KRT7 expression are significantly higher in cancerous tissue when compared to that of normal controls. This heightened KRT7 expression is linked to cancer progression, metastasis, and EMT. *In vitro* and *in vivo* results confirmed that KRT7 knockdown significantly impedes cancer advancement and metastasis while also enhancing sensitivity to chemotherapy in cancer cells, suggesting that KRT7 holds potential as a candidate for cancer treatment. Nevertheless, additional evidence from both *in vitro* and *in vivo* is required to further support the efficacy of KRT7 inhibition in cancer treatment. Furthermore, our review highlights KRT7’s critical role in regulating the tumor microenvironment and immune response, where aberrant KRT7 expression is associated with a suppressive immune microenvironment. Additionally, we have demonstrated the potential of KRT7 as a diagnostic and prognostic indicator in a variety of cancers. These findings significantly contribute to KRT7 research and drug development by aiding in our comprehension of KRT7’s function in tumorigenesis and development, as well as its potential clinical utility.

## Author contributions

All authors contributed in various steps of preparation. All authors contributed to the article and approved the submitted version.

## Glossary

**Table:**

KRT8	Keratin 8
CK -7	cytokeratin-7
K7	keratin-7
EMT	epithelial-mesenchymal transition
PFS	progression-free survival
MSS	microsatellite stability
MSI	microsatellite instability
ACSS2	Acetyl-CoA synthetase-2
KRT20	keratin 20
CTC	circulating tumor cells
RC	radical cystectomy
AC	adenocarcinoma
ITG-1	inhibition of thrombin generation-1
MMP2	Matrix Metallopeptidase 2
SMAD2	SMAD Family Member 2
SNAI	Snail Family Transcriptional Repressor
Twist	Twist-related protein
FN	fibronectin
Vim	Vimentin
FAK	Focal Adhesion Kinase
ECM	extracellular matrix
PTKs	protein tyrosine kinases
m6A	N6-methyladenosine
METTL3	methyltransferase-like 3
LM	lung metastasis
HBXIP	hepatitis B X-interacting protein
KRT7-AS	Keratin7-antisense
CDS	coding sequence
FTO	fat mass and obesity-associated protein
IGF2BP1	insulin-like growth factor 2 mRNA-binding protein 1
RBRP	RNA-binding regulatory peptide
HuR	human antigen R
CRC	colorectal cancer
Fn	Fusobacterium nucleatum
TLR4	Toll-like receptor 4
ESCCs	esophageal squamous cell carcinomas
FOXA1	Forkhead box protein A1
SA	sennoside A
HCC	hepatocellular carcinoma
WT1	Wilms tumor protein 1
S100A2	S100 Calcium Binding Protein A2
EF	elongation factor
HAVCR2	hepatitis A virus cellular receptor 2
PD -1	programmed cell death protein 1
CAR	chimeric antigen receptor
LOXL2	lysyl oxidases-like 2
TIM3	T cell immunoglobulin mucin 3
Tregs	regulatory T cells
CTLs	cytotoxic T lymphocytes
TME	tumor microenvironment
TIICs	Tumor-infiltrating immune cells
MAIT	mucosa-associated invariant T
NK	natural killer
Tgd	gamma delta T
NKT	natural killer T
DCs	dendritic cells
ICC	intrahepatic cholangiocarcinoma
PBA	primary bladder adenocarcinoma
MCA	metastatic colon adenocarcinoma
pRCT	papillary renal tumor
MTSCC	mucinous tubular and spindle cell carcinoma
BC	bladder cancer
NBE	normal bladder epithelium
